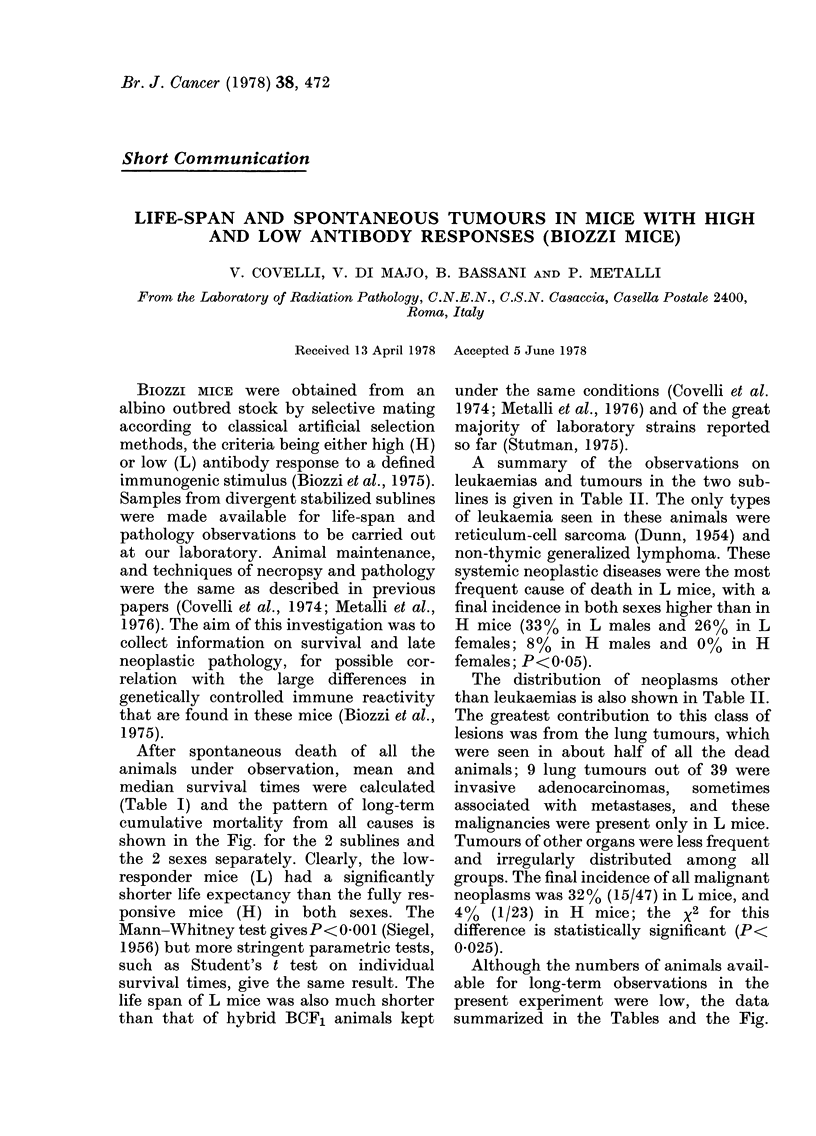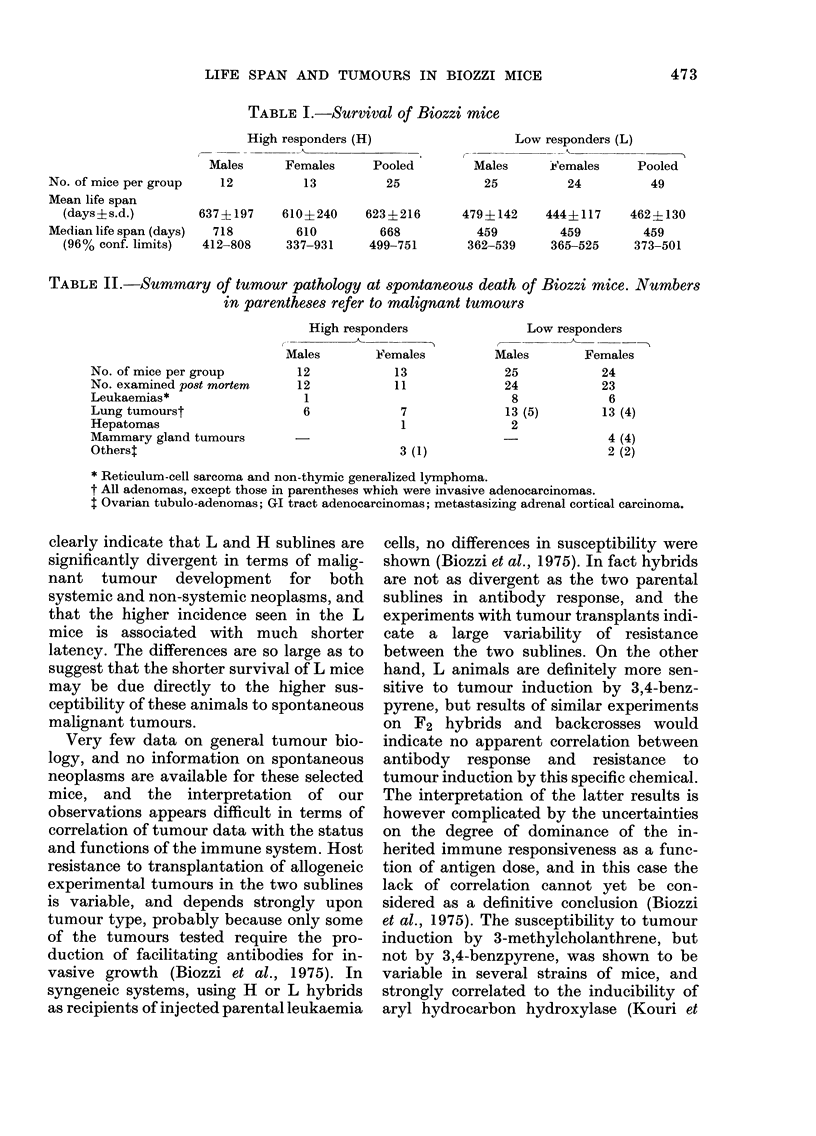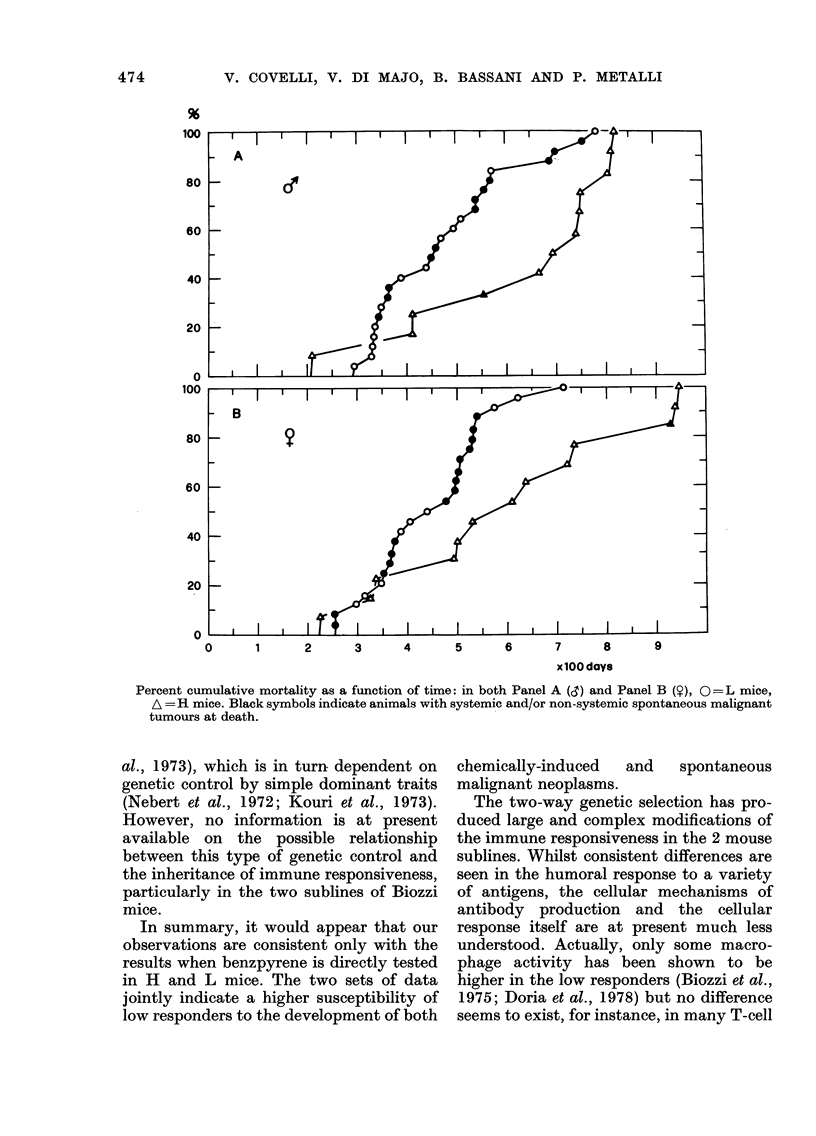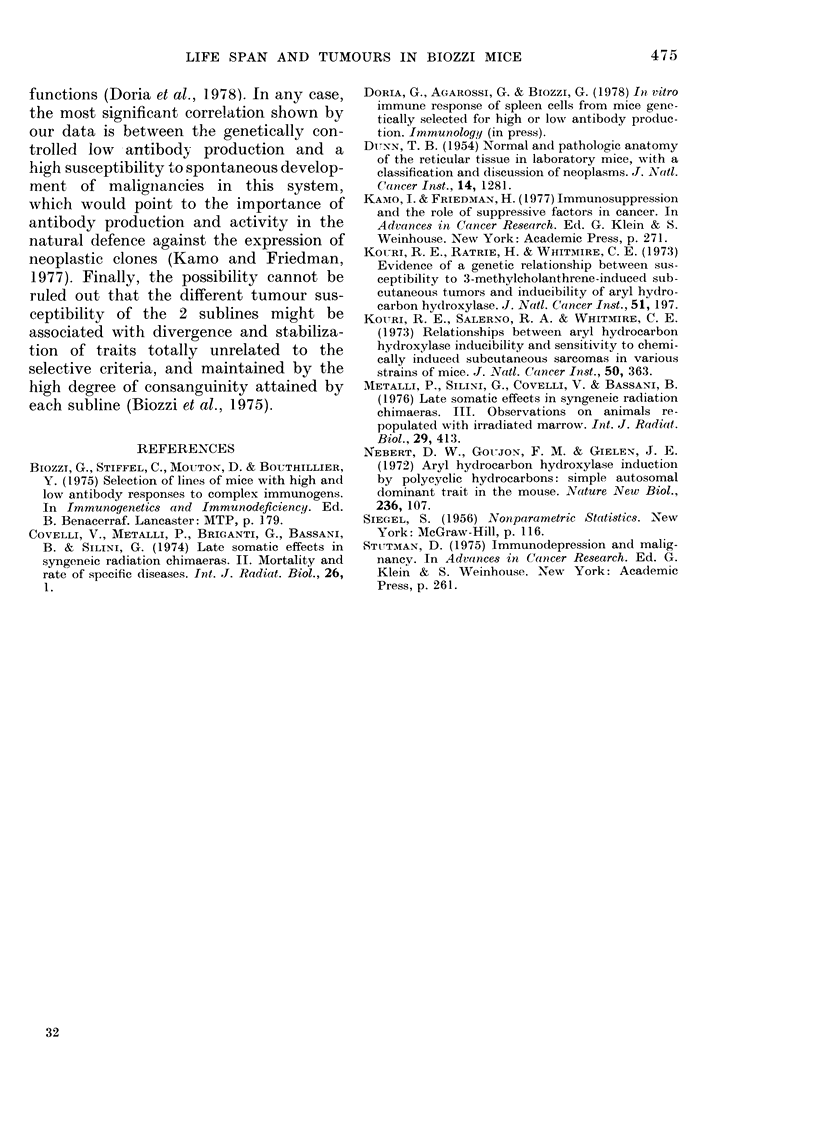# Life-span and spontaneous tumours in mice with high and low antibody responses (Biozzi mice).

**DOI:** 10.1038/bjc.1978.233

**Published:** 1978-09

**Authors:** V. Covelli, V. Di Majo, B. Bassani, P. Metalli


*Br. J. Cancer* (1978) **38**, 472

***Short Communication***

### LIFE-SPAN AND SPONTANEOUS TUMOURS IN MICE WITH HIGH AND LOW ANTIBODY RESPONSES (BIOZZI MICE)

V. COVELLI, V. DI MAJO, B. BASSANI AND P. METALLI

*From the Laboratory of Radiation Pathology, C.N.E.N., C.S.N. Casaccia, Casella Postale* 2400, *Roma, Italy*

Received 13 April 1978 Accepted 5 June 1978

BIOZZI MICE were obtained from an albino outbred stock by selective mating according to classical artificial selection methods, the criteria being either high (H) or low (L) antibody response to a defined immunogenic stimulus (Biozzi *et al.*, 1975). Samples from divergent stabilized sublines were made available for life-span and pathology observations to be carried out at our laboratory. Animal maintenance, and techniques of necropsy and pathology were the same as described in previous papers (Covelli *et al.*, 1974; Metalli *et al.*, 1976). The aim of this investigation was to collect information on survival and late neoplastic pathology, for possible correlation with the large differences in genetically controlled immune reactivity that are found in these mice (Biozzi *et al.*, 1975).

After spontaneous death of all the animals under observation, mean and median survival times were calculated (Table I) and the pattern of long-term cumulative mortality from all causes is shown in the Fig. for the 2 sublines and the 2 sexes separately. Clearly, the low-responder mice (L) had a significantly shorter life expectancy than the fully responsive mice (H) in both sexes. The Mann–Whitney test gives $P<0{\cdot}001$ (Siegel, 1956) but more stringent parametric tests, such as Student's *t* test on individual survival times, give the same result. The life span of L mice was also much shorter than that of hybrid $BCF_1$ animals kept under the same conditions (Covelli *et al.* 1974; Metalli *et al.*, 1976) and of the great majority of laboratory strains reported so far (Stutman, 1975).

A summary of the observations on leukaemias and tumours in the two sublines is given in Table II. The only types of leukaemia seen in these animals were reticulum-cell sarcoma (Dunn, 1954) and non-thymic generalized lymphoma. These systemic neoplastic diseases were the most frequent cause of death in L mice, with a final incidence in both sexes higher than in H mice (33% in L males and 26% in L females; 8% in H males and 0% in H females; $P<0{\cdot}05$).

The distribution of neoplasms other than leukaemias is also shown in Table II. The greatest contribution to this class of lesions was from the lung tumours, which were seen in about half of all the dead animals; 9 lung tumours out of 39 were invasive adenocarcinomas, sometimes associated with metastases, and these malignancies were present only in L mice. Tumours of other organs were less frequent and irregularly distributed among all groups. The final incidence of all malignant neoplasms was 32% (15/47) in L mice, and 4% (1/23) in H mice; the $\chi^2$ for this difference is statistically significant ($P<0{\cdot}025$).

Although the numbers of animals available for long-term observations in the present experiment were low, the data summarized in the Tables and the Fig.

TABLE I.—*Survival of Biozzi mice*

| | High responders (H) | | | Low responders (L) | | |
|---|---|---|---|---|---|---|
| | Males | Females | Pooled | Males | Females | Pooled |
| No. of mice per group | 12 | 13 | 25 | 25 | 24 | 49 |
| Mean life span (days±s.d.) | 637±197 | 610±240 | 623±216 | 479±142 | 444±117 | 462±130 |
| Median life span (days) | 718 | 610 | 668 | 459 | 459 | 459 |
| (96% conf. limits) | 412–808 | 337–931 | 499–751 | 362–539 | 365–525 | 373–501 |

TABLE II.—*Summary of tumour pathology at spontaneous death of Biozzi mice. Numbers in parentheses refer to malignant tumours*

| | High responders | | Low responders | |
|---|---|---|---|---|
| | Males | Females | Males | Females |
| No. of mice per group | 12 | 13 | 25 | 24 |
| No. examined *post mortem* | 12 | 11 | 24 | 23 |
| Leukaemias* | 1 | | 8 | 6 |
| Lung tumours† | 6 | 7 | 13 (5) | 13 (4) |
| Hepatomas | | 1 | 2 | |
| Mammary gland tumours | — | | — | 4 (4) |
| Others‡ | | 3 (1) | | 2 (2) |

* Reticulum-cell sarcoma and non-thymic generalized lymphoma.
† All adenomas, except those in parentheses which were invasive adenocarcinomas.
‡ Ovarian tubulo-adenomas; GI tract adenocarcinomas; metastasizing adrenal cortical carcinoma.

clearly indicate that L and H sublines are significantly divergent in terms of malignant tumour development for both systemic and non-systemic neoplasms, and that the higher incidence seen in the L mice is associated with much shorter latency. The differences are so large as to suggest that the shorter survival of L mice may be due directly to the higher susceptibility of these animals to spontaneous malignant tumours.

Very few data on general tumour biology, and no information on spontaneous neoplasms are available for these selected mice, and the interpretation of our observations appears difficult in terms of correlation of tumour data with the status and functions of the immune system. Host resistance to transplantation of allogeneic experimental tumours in the two sublines is variable, and depends strongly upon tumour type, probably because only some of the tumours tested require the production of facilitating antibodies for invasive growth (Biozzi *et al.*, 1975). In syngeneic systems, using H or L hybrids as recipients of injected parental leukaemia cells, no differences in susceptibility were shown (Biozzi *et al.*, 1975). In fact hybrids are not as divergent as the two parental sublines in antibody response, and the experiments with tumour transplants indicate a large variability of resistance between the two sublines. On the other hand, L animals are definitely more sensitive to tumour induction by 3,4-benzpyrene, but results of similar experiments on $F_2$ hybrids and backcrosses would indicate no apparent correlation between antibody response and resistance to tumour induction by this specific chemical. The interpretation of the latter results is however complicated by the uncertainties on the degree of dominance of the inherited immune responsiveness as a function of antigen dose, and in this case the lack of correlation cannot yet be considered as a definitive conclusion (Biozzi *et al.*, 1975). The susceptibility to tumour induction by 3-methylcholanthrene, but not by 3,4-benzpyrene, was shown to be variable in several strains of mice, and strongly correlated to the inducibility of aryl hydrocarbon hydroxylase (Kouri *et*

Percent cumulative mortality as a function of time: in both Panel A (♂) and Panel B (♀), ○ = L mice, △ = H mice. Black symbols indicate animals with systemic and/or non-systemic spontaneous malignant tumours at death.

*al.*, 1973), which is in turn dependent on genetic control by simple dominant traits (Nebert *et al.*, 1972; Kouri *et al.*, 1973). However, no information is at present available on the possible relationship between this type of genetic control and the inheritance of immune responsiveness, particularly in the two sublines of Biozzi mice.

In summary, it would appear that our observations are consistent only with the results when benzpyrene is directly tested in H and L mice. The two sets of data jointly indicate a higher susceptibility of low responders to the development of both chemically-induced and spontaneous malignant neoplasms.

The two-way genetic selection has produced large and complex modifications of the immune responsiveness in the 2 mouse sublines. Whilst consistent differences are seen in the humoral response to a variety of antigens, the cellular mechanisms of antibody production and the cellular response itself are at present much less understood. Actually, only some macrophage activity has been shown to be higher in the low responders (Biozzi *et al.*, 1975; Doria *et al.*, 1978) but no difference seems to exist, for instance, in many T-cell functions (Doria *et al.*, 1978). In any case, the most significant correlation shown by our data is between the genetically controlled low antibody production and a high susceptibility to spontaneous development of malignancies in this system, which would point to the importance of antibody production and activity in the natural defence against the expression of neoplastic clones (Kamo and Friedman, 1977). Finally, the possibility cannot be ruled out that the different tumour susceptibility of the 2 sublines might be associated with divergence and stabilization of traits totally unrelated to the selective criteria, and maintained by the high degree of consanguinity attained by each subline (Biozzi *et al.*, 1975).

### REFERENCES

BIOZZI, G., STIFFEL, C., MOUTON, D. & BOUTHILLIER, Y. (1975) Selection of lines of mice with high and low antibody responses to complex immunogens. In *Immunogenetics and Immunodeficiency*. Ed. B. Benacerraf. Lancaster: MTP, p. 179.

COVELLI, V., METALLI, P., BRIGANTI, G., BASSANI, B. & SILINI, G. (1974) Late somatic effects in syngeneic radiation chimaeras. II. Mortality and rate of specific diseases. *Int. J. Radiat. Biol.*, **26**, 1.

DORIA, G., AGAROSSI, G. & BIOZZI, G. (1978) *In vitro* immune response of spleen cells from mice genetically selected for high or low antibody production. *Immunology* (in press).

DUNN, T. B. (1954) Normal and pathologic anatomy of the reticular tissue in laboratory mice, with a classification and discussion of neoplasms. *J. Natl. Cancer Inst.*, **14**, 1281.

KAMO, I. & FRIEDMAN, H. (1977) Immunosuppression and the role of suppressive factors in cancer. In *Advances in Cancer Research*. Ed. G. Klein & S. Weinhouse. New York: Academic Press, p. 271.

KOURI, R. E., RATRIE, H. & WHITMIRE, C. E. (1973) Evidence of a genetic relationship between susceptibility to 3-methylcholanthrene-induced subcutaneous tumors and inducibility of aryl hydrocarbon hydroxylase. *J. Natl. Cancer Inst.*, **51**, 197.

KOURI, R. E., SALERNO, R. A. & WHITMIRE, C. E. (1973) Relationships between aryl hydrocarbon hydroxylase inducibility and sensitivity to chemically induced subcutaneous sarcomas in various strains of mice. *J. Natl. Cancer Inst.*, **50**, 363.

METALLI, P., SILINI, G., COVELLI, V. & BASSANI, B. (1976) Late somatic effects in syngeneic radiation chimaeras. III. Observations on animals repopulated with irradiated marrow. *Int. J. Radiat. Biol.*, **29**, 413.

NEBERT, D. W., GOUJON, F. M. & GIELEN, J. E. (1972) Aryl hydrocarbon hydroxylase induction by polycyclic hydrocarbons: simple autosomal dominant trait in the mouse. *Nature New Biol.*, **236**, 107.

SIEGEL, S. (1956) *Nonparametric Statistics*. New York: McGraw-Hill, p. 116.

STUTMAN, D. (1975) Immunodepression and malignancy. In *Advances in Cancer Research*. Ed. G. Klein & S. Weinhouse. New York: Academic Press, p. 261.